# *rac*-4-Amino-1-(2-benzoyl-1-phenyl­eth­yl)-3-methyl-1*H*-1,2,4-triazole-5(4*H*)-thione

**DOI:** 10.1107/S1600536810054255

**Published:** 2011-01-12

**Authors:** Wei Wang, Yan Gao, Zuo-bing Xiao, Hong-guo Yao, Jing-jing Zhang

**Affiliations:** a School of Perfume and Aroma Technology, Shanghai Institute of Technology, Shanghai 200235, People’s Republic of China; b School of Chemical Engineering, University of Science and Technology LiaoNing, Anshan 114051, People’s Republic of China

## Abstract

The title compound, C_18_H_18_N_4_OS, has an almost planar 1,2,4-triazole ring [r.m.s. deviation = 0.0036 (2) Å], which makes dihedral angles of 78.5 (2) and 77.6 (11)° with the two phenyl rings. An intra­molecular N—H⋯S inter­action occurs. In the crystal, mol­ecules are linked by an inter­molecular three-centre N—H⋯(O,S) cyclic hydrogen-bonding inter­action.

## Related literature

For standard bond lengths, see: Allen *et al.* (1987 ▶). For the crystal structures of isomers of the title compound, see: Özel Güven *et al.* (2008*a* ▶,*b* ▶). For the pharmacological properties of triazole compounds, see: Paulvannan *et al.* (2001 ▶); Wahbi *et al.* (1995 ▶).
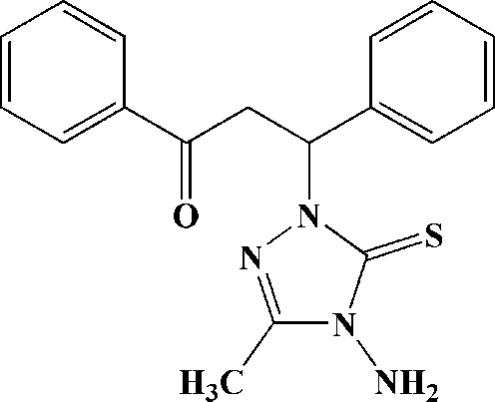


## Experimental

### 

#### Crystal data

C_18_H_18_N_4_OS
*M* *_r_* = 338.42
Orthorhombic, 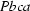

*a* = 17.604 (4) Å
*b* = 10.199 (2) Å
*c* = 19.241 (4) Å
*V* = 3454.5 (13) Å^3^
*Z* = 8
Mo *K*α radiation
μ = 0.20 mm^−1^
*T* = 293 K
0.24 × 0.22 × 0.10 mm

#### Data collection

Rigaku Saturn CCD area-detector diffractometer
Absorption correction: multi-scan (*CrystalClear*; Rigaku/MSC, 2005 ▶) *T*_min_ = 0.954, *T*_max_ = 0.980
25430 measured reflections
3040 independent reflections
2527 reflections with *I* > 2σ(*I*)
*R*_int_ = 0.057

#### Refinement

*R*[*F* ^2^ > 2σ(*F* ^2^)] = 0.055
*wR*(*F* ^2^) = 0.137
*S* = 1.13
3040 reflections
227 parameters
3 restraints
H atoms treated by a mixture of independent and constrained refinement
Δρ_max_ = 0.39 e Å^−3^
Δρ_min_ = −0.29 e Å^−3^

### 

Data collection: *CrystalClear* (Rigaku/MSC, 2005 ▶); cell refinement: *CrystalClear*; data reduction: *CrystalClear*; program(s) used to solve structure: *SHELXS97* (Sheldrick, 2008 ▶); program(s) used to refine structure: *SHELXL97* (Sheldrick, 2008 ▶); molecular graphics: *SHELXTL* (Sheldrick, 2008 ▶); software used to prepare material for publication: *SHELXTL*.

## Supplementary Material

Crystal structure: contains datablocks global, I. DOI: 10.1107/S1600536810054255/zs2086sup1.cif

Structure factors: contains datablocks I. DOI: 10.1107/S1600536810054255/zs2086Isup2.hkl

Additional supplementary materials:  crystallographic information; 3D view; checkCIF report

## Figures and Tables

**Table 1:** Hydrogen-bond geometry (Å, °)

*D*—H⋯*A*	*D*—H	H⋯*A*	*D*⋯*A*	*D*—H⋯*A*
N1—H1*A*⋯O1^i^	0.90 (2)	2.47 (2)	3.121 (3)	129 (2)
N1—H1*A*⋯S1	0.90 (2)	2.65 (3)	3.195 (2)	120 (2)
N1—H1*B*⋯S1^i^	0.90 (2)	2.45 (1)	3.340 (3)	172 (2)

Symmetry code: (i) 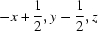.

**Fig. 1.**
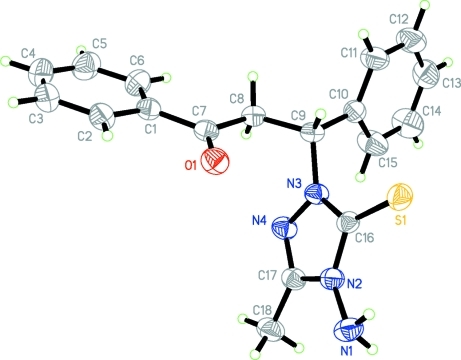
View of the molecule of (I) showing the atom-labelling scheme. Displacement ellipsoids are drawn at the 35% probability level.

## supplementary crystallographic information

### Comment

Functionalized 1,2,4-triazole derivatives are biologically interesting molecules and their chemistry is receiving considerable attention due to antihypertensive, antifungal and antibacterial properties (Paulvannan *et al.*, 2001; Wahbi *et al.*, 1995). Some crystal structures of 1*H*-1,2,4-triazole ring-containing ether derivatives have been reported recently (Özel Güven, *et al.*, 2008**a*, b*). Here we report the synthesis and crystal structure of the title compound, *rac*-4-amino-1-[(1,3-diphenylpropan-1-one)-3-yl]-3-methyl-1*H*-1,2,4-triazole-5(4*H*)-thione (I) (Fig. 1).

The bond lengths and angles in (I) are found to have normal values (Allen *et al.*, 1987). The 1,2,4-triazole ring (N2/C16/N3/N4/C17) is essentially planar with an r.m.s. deviation of 0.0036 (2)Å and a maximum deviation of 0.0057 (2)Å for atom N3. The two phenyl rings are inclined with respect to the 1,2,4-triazole ring [dihedral angles of 101.5 (2)° (C1–C6) and 102.4 (11)° (C10—C15)] with a dihedral angle between the two phenyl rings of 97.6°, which indicates that they are almost mutually perpendicular. In the crystal structure there is an intramolecular N—H···S interaction and the molecules are linked by an intermolecular three-centre N—H···O, N—H···S cyclic hydrogen-bonding interaction (Table 1).

### Experimental

The title compound was synthesized by the reaction of 1,3-diphenyl-2-propen-1-one (chalcone) (2.0 mmol) with 4-amino-1-methyl-4*H*-1,2,4-triazole-5-thiol (2.0 mmol) in ethanol. The reaction progress was monitored *via* TLC. The resulting precipitate was filtered off, washed with cold ethanol, dried and purified to give the target product as a colorless solid in 90% yield. Crystals of (I) suitable for single-crystal X-ray analysis were grown by slow evaporation of a solution in chloroform-ethanol (1:1).

### Refinement

The H atoms attached to N atoms were located in a difference electron density map and the atomic coordinates and isotropic displacement parameters were allowed to refine freely. Other H atoms were positioned geometrically and refined as riding with (C—H = 0.93–0.97 Å) and with *U*_iso_(H) = 1.2*U*_eq_(parent).

### Crystal data

**Table:**

C_18_H_18_N_4_OS	*F*(000) = 1424
*M**_r_* = 338.42	*D*_x_ = 1.301 Mg m^−^^3^
Orthorhombic, *P**b**c**a*	Mo *K*α radiation, λ = 0.71073 Å
Hall symbol: -P 2ac 2ab	Cell parameters from 7030 reflections
*a* = 17.604 (4) Å	θ = 2.1–27.9°
*b* = 10.199 (2) Å	µ = 0.20 mm^−^^1^
*c* = 19.241 (4) Å	*T* = 293 K
*V* = 3454.5 (13) Å^3^	Prism, colorless
*Z* = 8	0.24 × 0.22 × 0.10 mm

### Data collection

**Table:**

Rigaku Saturn CCD area-detector diffractometer	3040 independent reflections
Radiation source: rotating anode X-ray tube	2527 reflections with *I* > 2σ(*I*)
confocal	*R*_int_ = 0.057
Detector resolution: 7.31 pixels mm^-1^	θ_max_ = 25.0°, θ_min_ = 2.1°
φ and ω scans	*h* = −20→20
Absorption correction: multi-scan (*CrystalClear*; Rigaku/MSC, 2005)	*k* = −12→11
*T*_min_ = 0.954, *T*_max_ = 0.980	*l* = −22→22
25430 measured reflections	

### Refinement

**Table:**

Refinement on *F*^2^	Secondary atom site location: difference Fourier map
Least-squares matrix: full	Hydrogen site location: inferred from neighbouring sites
*R*[*F*^2^ > 2σ(*F*^2^)] = 0.055	H atoms treated by a mixture of independent and constrained refinement
*wR*(*F*^2^) = 0.137	*w* = 1/[σ^2^(*F*_o_^2^) + (0.060*P*)^2^ + 1.1394*P*] where *P* = (*F*_o_^2^ + 2*F*_c_^2^)/3
*S* = 1.13	(Δ/σ)_max_ = 0.001
3040 reflections	Δρ_max_ = 0.39 e Å^−^^3^
227 parameters	Δρ_min_ = −0.29 e Å^−^^3^
3 restraints	Extinction correction: *SHELXL97* (Sheldrick, 2008), Fc^*^=kFc[1+0.001xFc^2^λ^3^/sin(2θ)]^-1/4^
Primary atom site location: structure-invariant direct methods	Extinction coefficient: 0.0148 (12)

### Special details

**Table:**

Geometry. All e.s.d.'s (except the e.s.d. in the dihedral angle between two l.s. planes) are estimated using the full covariance matrix. The cell e.s.d.'s are taken into account individually in the estimation of e.s.d.'s in distances, angles and torsion angles; correlations between e.s.d.'s in cell parameters are only used when they are defined by crystal symmetry. An approximate (isotropic) treatment of cell e.s.d.'s is used for estimating e.s.d.'s involving l.s. planes.
Refinement. Refinement of *F*^2^ against ALL reflections. The weighted *R*-factor *wR* and goodness of fit *S* are based on *F*^2^, conventional *R*-factors *R* are based on *F*, with *F* set to zero for negative *F*^2^. The threshold expression of *F*^2^ > σ(*F*^2^) is used only for calculating *R*-factors(gt) *etc*. and is not relevant to the choice of reflections for refinement. *R*-factors based on *F*^2^ are statistically about twice as large as those based on *F*, and *R*- factors based on ALL data will be even larger.

### Fractional atomic coordinates and isotropic or equivalent isotropic displacement parameters (Å^2^)

**Table:**

	*x*	*y*	*z*	*U*_iso_*/*U*_eq_	
S1	0.20040 (3)	0.45904 (6)	0.42466 (4)	0.0526 (2)	
O1	0.38811 (10)	0.67534 (18)	0.34992 (8)	0.0550 (5)	
N1	0.28460 (14)	0.2386 (2)	0.33526 (11)	0.0550 (6)	
H1A	0.2383 (9)	0.276 (2)	0.3333 (17)	0.101 (13)*	
H1B	0.2847 (14)	0.1601 (16)	0.3563 (14)	0.074 (10)*	
N2	0.32401 (11)	0.31994 (19)	0.38230 (9)	0.0437 (5)	
N3	0.35336 (10)	0.47896 (19)	0.44806 (10)	0.0427 (5)	
N4	0.42070 (11)	0.4176 (2)	0.43219 (10)	0.0494 (5)	
C1	0.49061 (13)	0.8173 (2)	0.37434 (12)	0.0472 (6)	
C2	0.49301 (16)	0.8628 (3)	0.30604 (13)	0.0598 (7)	
H2	0.4568	0.8345	0.2742	0.072*	
C3	0.54873 (19)	0.9494 (3)	0.28535 (17)	0.0732 (9)	
H3	0.5496	0.9796	0.2398	0.088*	
C4	0.60258 (19)	0.9911 (3)	0.33125 (19)	0.0778 (9)	
H4	0.6403	1.0486	0.3167	0.093*	
C5	0.60126 (18)	0.9482 (3)	0.39905 (19)	0.0754 (9)	
H5	0.6379	0.9771	0.4303	0.091*	
C6	0.54545 (15)	0.8625 (3)	0.42060 (14)	0.0606 (7)	
H6	0.5445	0.8345	0.4666	0.073*	
C7	0.43012 (13)	0.7222 (2)	0.39396 (12)	0.0439 (6)	
C8	0.42195 (13)	0.6852 (2)	0.46984 (11)	0.0438 (6)	
H8A	0.4203	0.7649	0.4974	0.053*	
H8B	0.4666	0.6363	0.4839	0.053*	
C9	0.35175 (12)	0.6038 (2)	0.48612 (11)	0.0421 (6)	
H9	0.3075	0.6531	0.4696	0.051*	
C10	0.34152 (13)	0.5820 (3)	0.56370 (12)	0.0451 (6)	
C11	0.30483 (17)	0.6765 (3)	0.60210 (14)	0.0659 (8)	
H11	0.2866	0.7518	0.5804	0.079*	
C12	0.2949 (2)	0.6597 (4)	0.67341 (15)	0.0805 (10)	
H12	0.2703	0.7239	0.6994	0.097*	
C13	0.32127 (19)	0.5483 (4)	0.70528 (15)	0.0757 (9)	
H13	0.3153	0.5376	0.7530	0.091*	
C14	0.3560 (2)	0.4543 (4)	0.66734 (16)	0.0847 (11)	
H14	0.3729	0.3781	0.6890	0.102*	
C15	0.36657 (19)	0.4705 (3)	0.59659 (15)	0.0734 (9)	
H15	0.3908	0.4053	0.5711	0.088*	
C16	0.29294 (13)	0.4212 (2)	0.41909 (11)	0.0399 (5)	
C17	0.40078 (14)	0.3207 (2)	0.39139 (12)	0.0463 (6)	
C18	0.45268 (16)	0.2216 (3)	0.36137 (14)	0.0629 (7)	
H18A	0.5043	0.2491	0.3679	0.094*	
H18B	0.4425	0.2124	0.3126	0.094*	
H18C	0.4448	0.1389	0.3841	0.094*	

### Atomic displacement parameters (Å^2^)

**Table:**

	*U*^11^	*U*^22^	*U*^33^	*U*^12^	*U*^13^	*U*^23^
S1	0.0435 (4)	0.0467 (4)	0.0676 (5)	−0.0016 (3)	0.0024 (3)	−0.0004 (3)
O1	0.0561 (10)	0.0622 (12)	0.0466 (9)	−0.0008 (9)	−0.0050 (8)	−0.0057 (8)
N1	0.0718 (16)	0.0453 (13)	0.0480 (12)	−0.0055 (12)	−0.0067 (11)	−0.0062 (11)
N2	0.0536 (12)	0.0386 (11)	0.0389 (10)	0.0011 (9)	0.0004 (9)	−0.0008 (8)
N3	0.0411 (10)	0.0400 (11)	0.0471 (10)	0.0049 (9)	−0.0014 (8)	−0.0054 (9)
N4	0.0436 (11)	0.0500 (12)	0.0546 (12)	0.0091 (10)	−0.0012 (9)	−0.0044 (10)
C1	0.0458 (13)	0.0443 (14)	0.0516 (13)	0.0069 (11)	0.0028 (11)	−0.0015 (11)
C2	0.0646 (17)	0.0625 (18)	0.0523 (15)	0.0018 (14)	0.0085 (12)	−0.0003 (13)
C3	0.088 (2)	0.067 (2)	0.0651 (18)	−0.0005 (17)	0.0245 (17)	0.0058 (15)
C4	0.072 (2)	0.065 (2)	0.096 (3)	−0.0128 (17)	0.0255 (19)	−0.0014 (19)
C5	0.0619 (18)	0.069 (2)	0.095 (2)	−0.0124 (16)	−0.0030 (17)	−0.0063 (18)
C6	0.0596 (16)	0.0580 (17)	0.0643 (16)	−0.0044 (14)	−0.0049 (13)	0.0019 (14)
C7	0.0435 (12)	0.0415 (14)	0.0468 (13)	0.0089 (10)	−0.0006 (11)	−0.0033 (11)
C8	0.0471 (13)	0.0409 (13)	0.0436 (12)	0.0029 (10)	−0.0041 (10)	−0.0034 (11)
C9	0.0439 (12)	0.0376 (13)	0.0449 (12)	0.0052 (10)	−0.0025 (10)	−0.0056 (10)
C10	0.0422 (12)	0.0485 (15)	0.0447 (13)	−0.0018 (11)	−0.0007 (10)	−0.0036 (11)
C11	0.090 (2)	0.0536 (17)	0.0543 (15)	0.0102 (15)	0.0103 (14)	−0.0033 (13)
C12	0.112 (3)	0.074 (2)	0.0549 (17)	0.001 (2)	0.0184 (17)	−0.0141 (17)
C13	0.094 (2)	0.089 (3)	0.0440 (15)	−0.0094 (19)	0.0060 (15)	0.0011 (16)
C14	0.113 (3)	0.087 (3)	0.0544 (17)	0.024 (2)	−0.0020 (17)	0.0179 (17)
C15	0.097 (2)	0.069 (2)	0.0545 (16)	0.0295 (18)	0.0053 (16)	0.0062 (15)
C16	0.0474 (13)	0.0335 (12)	0.0387 (11)	−0.0001 (10)	0.0002 (9)	0.0024 (10)
C17	0.0534 (14)	0.0430 (14)	0.0426 (12)	0.0083 (11)	0.0018 (11)	0.0015 (11)
C18	0.0720 (18)	0.0550 (17)	0.0616 (16)	0.0157 (14)	0.0100 (14)	−0.0065 (14)

### Geometric parameters (Å, °)

**Table:**

S1—C16	1.678 (2)	C6—H6	0.9300
O1—C7	1.222 (3)	C7—C8	1.515 (3)
N1—N2	1.410 (3)	C8—C9	1.521 (3)
N1—H1A	0.900 (10)	C8—H8A	0.9700
N1—H1B	0.897 (10)	C8—H8B	0.9700
N2—C17	1.363 (3)	C9—C10	1.520 (3)
N2—C16	1.366 (3)	C9—H9	0.9800
N3—C16	1.338 (3)	C10—C15	1.375 (4)
N3—N4	1.375 (3)	C10—C11	1.375 (4)
N3—C9	1.469 (3)	C11—C12	1.394 (4)
N4—C17	1.310 (3)	C11—H11	0.9300
C1—C6	1.392 (3)	C12—C13	1.372 (5)
C1—C2	1.394 (3)	C12—H12	0.9300
C1—C7	1.489 (3)	C13—C14	1.351 (5)
C2—C3	1.379 (4)	C13—H13	0.9300
C2—H2	0.9300	C14—C15	1.384 (4)
C3—C4	1.363 (4)	C14—H14	0.9300
C3—H3	0.9300	C15—H15	0.9300
C4—C5	1.376 (5)	C17—C18	1.480 (3)
C4—H4	0.9300	C18—H18A	0.9600
C5—C6	1.379 (4)	C18—H18B	0.9600
C5—H5	0.9300	C18—H18C	0.9600
			
N2—N1—H1A	103 (2)	N3—C9—C10	111.4 (2)
N2—N1—H1B	103.5 (19)	N3—C9—C8	110.77 (17)
H1A—N1—H1B	113.5 (16)	C10—C9—C8	112.23 (18)
C17—N2—C16	109.03 (19)	N3—C9—H9	107.4
C17—N2—N1	125.0 (2)	C10—C9—H9	107.4
C16—N2—N1	125.5 (2)	C8—C9—H9	107.4
C16—N3—N4	113.11 (19)	C15—C10—C11	118.9 (2)
C16—N3—C9	125.05 (18)	C15—C10—C9	122.3 (2)
N4—N3—C9	121.46 (18)	C11—C10—C9	118.7 (2)
C17—N4—N3	104.22 (19)	C10—C11—C12	120.1 (3)
C6—C1—C2	118.2 (2)	C10—C11—H11	119.9
C6—C1—C7	123.3 (2)	C12—C11—H11	119.9
C2—C1—C7	118.5 (2)	C13—C12—C11	119.9 (3)
C3—C2—C1	120.4 (3)	C13—C12—H12	120.0
C3—C2—H2	119.8	C11—C12—H12	120.0
C1—C2—H2	119.8	C14—C13—C12	120.0 (3)
C4—C3—C2	120.5 (3)	C14—C13—H13	120.0
C4—C3—H3	119.8	C12—C13—H13	120.0
C2—C3—H3	119.8	C13—C14—C15	120.5 (3)
C3—C4—C5	120.2 (3)	C13—C14—H14	119.7
C3—C4—H4	119.9	C15—C14—H14	119.7
C5—C4—H4	119.9	C10—C15—C14	120.5 (3)
C4—C5—C6	119.9 (3)	C10—C15—H15	119.7
C4—C5—H5	120.1	C14—C15—H15	119.7
C6—C5—H5	120.1	N3—C16—N2	103.33 (19)
C5—C6—C1	120.8 (3)	N3—C16—S1	130.09 (18)
C5—C6—H6	119.6	N2—C16—S1	126.58 (18)
C1—C6—H6	119.6	N4—C17—N2	110.3 (2)
O1—C7—C1	120.8 (2)	N4—C17—C18	125.7 (2)
O1—C7—C8	120.9 (2)	N2—C17—C18	123.9 (2)
C1—C7—C8	118.3 (2)	C17—C18—H18A	109.5
C7—C8—C9	114.30 (19)	C17—C18—H18B	109.5
C7—C8—H8A	108.7	H18A—C18—H18B	109.5
C9—C8—H8A	108.7	C17—C18—H18C	109.5
C7—C8—H8B	108.7	H18A—C18—H18C	109.5
C9—C8—H8B	108.7	H18B—C18—H18C	109.5
H8A—C8—H8B	107.6		
			
C16—N3—N4—C17	1.0 (3)	N3—C9—C10—C11	−150.2 (2)
C9—N3—N4—C17	−172.2 (2)	C8—C9—C10—C11	84.9 (3)
C6—C1—C2—C3	0.4 (4)	C15—C10—C11—C12	1.3 (4)
C7—C1—C2—C3	−179.0 (2)	C9—C10—C11—C12	−179.7 (3)
C1—C2—C3—C4	0.5 (4)	C10—C11—C12—C13	−0.4 (5)
C2—C3—C4—C5	−0.8 (5)	C11—C12—C13—C14	−0.9 (5)
C3—C4—C5—C6	0.3 (5)	C12—C13—C14—C15	1.3 (6)
C4—C5—C6—C1	0.7 (5)	C11—C10—C15—C14	−0.9 (5)
C2—C1—C6—C5	−1.0 (4)	C9—C10—C15—C14	−179.9 (3)
C7—C1—C6—C5	178.5 (3)	C13—C14—C15—C10	−0.4 (6)
C6—C1—C7—O1	−172.5 (2)	N4—N3—C16—N2	−1.0 (2)
C2—C1—C7—O1	7.0 (3)	C9—N3—C16—N2	171.9 (2)
C6—C1—C7—C8	7.6 (3)	N4—N3—C16—S1	178.93 (17)
C2—C1—C7—C8	−173.0 (2)	C9—N3—C16—S1	−8.1 (4)
O1—C7—C8—C9	−8.5 (3)	C17—N2—C16—N3	0.7 (2)
C1—C7—C8—C9	171.4 (2)	N1—N2—C16—N3	−171.6 (2)
C16—N3—C9—C10	93.1 (3)	C17—N2—C16—S1	−179.32 (18)
N4—N3—C9—C10	−94.6 (2)	N1—N2—C16—S1	8.4 (3)
C16—N3—C9—C8	−141.2 (2)	N3—N4—C17—N2	−0.6 (3)
N4—N3—C9—C8	31.1 (3)	N3—N4—C17—C18	−178.0 (2)
C7—C8—C9—N3	61.1 (2)	C16—N2—C17—N4	−0.1 (3)
C7—C8—C9—C10	−173.70 (19)	N1—N2—C17—N4	172.2 (2)
N3—C9—C10—C15	28.7 (3)	C16—N2—C17—C18	177.4 (2)
C8—C9—C10—C15	−96.1 (3)	N1—N2—C17—C18	−10.3 (4)

### Hydrogen-bond geometry (Å, °)

**Table:**

*D*—H···*A*	*D*—H	H···*A*	*D*···*A*	*D*—H···*A*
N1—H1A···O1^i^	0.90 (2)	2.47 (2)	3.121 (3)	129.(2)
N1—H1A···S1	0.90 (2)	2.65 (3)	3.195 (2)	120 (2)
N1—H1B···S1^i^	0.90 (2)	2.45 (1)	3.340 (3)	172.(2)

Symmetry codes: (i) −*x*+1/2, *y*−1/2, *z*.
